# Clinical, Echocardiographic, and Longitudinal Characteristics Associated With Heart Failure With Improved Ejection Fraction

**DOI:** 10.1016/j.amjcard.2023.10.086

**Published:** 2023-11-02

**Authors:** Erick Romero, Alexander Francois Baltodano, Paulo Rocha, Camryn Sellers-Porter, Dev Jaydeep Patel, Saad Soroya, Julie Bidwell, Imo Ebong, Michael Gibson, David A. Liem, Shirin Jimenez, Heejung Bang, Padmini Sirish, Nipavan Chiamvimonvat, Javier E. Lopez, Martin Cadeiras

**Affiliations:** a Division of Cardiovascular Medicine, UC Davis Medical Center, Sacramento, California; b School of Medicine, University of California Davis, Sacramento, California; c Betty Irene Moore School of Nursing, Family Caregiving Institute, University of California Davis, Sacramento, California; d Division of Biostatistics, Department of Public Health Sciences, University of California Davis, Davis, California; e Division of Cardiovascular Medicine, Department of Internal Medicine, University of California Davis, Davis, California.

**Keywords:** echocardiogram, HFimpEF, HFrEF, trajectories

## Abstract

Heart failure with improved ejection fraction (HFimpEF) has better outcomes than HF with reduced EF (HFrEF). However, factors contributing to HFimpEF remain unclear. This study aimed to evaluate clinical and longitudinal characteristics associated with subsequent HFimpEF. This was a single-center retrospective HFrEF cohort study. Data were collected from 2014 to 2022. Patients with HFrEF were identified using International Classification of Diseases codes, echocardiographic data, and natriuretic peptide levels. The main end points were HFimpEF (defined as EF >40% at ≥3 months with ≥10% increase) and mortality. Cox proportional hazards and mixed effects models were used for analyses. The study included 1,307 patients with HFrEF with a median follow-up of 16.3 months (interquartile range 8.0 to 30.6). The median age was 65 years; 68% were male whereas 57% were White. On follow-up, 38.7% (n = 506) developed HFimpEF, whereas 61.3% (n = 801) had persistent HFrEF. A multivariate Cox regression model identified gender, race, co-morbidities, echocardiographic, and natriuretic peptide as significant covariates of HFimpEF (p <0.05). The HFimpEF group had better survival compared with the persistent HFrEF group (p <0.001). Echocardiographic and laboratory trajectories differed between groups. In this HFrEF cohort, 38.7% transitioned to HFimpEF and approximately 50% met the definition within the first 12 months. In a HFimpEF model, gender, co-morbidities, echocardiographic parameters, and natriuretic peptide were associated with subsequent HFimpEF. The model has the potential to identify patients at risk of subsequent persistent or improved HFrEF, thus informing the design and implementation of targeted quality-of-care improvement interventions.

For a subset of patients with heart failure with reduced ejection fraction (HFrEF), some improvement in EF occurs. To be formally classified as HF with improved EF (HFimpEF), however, the patients must have a baseline left ventricular EF (LVEF) of ≤40% and subsequent LVEF >40% at ≥3 months, with ≥10% absolute increase.^1–3^ The prognosis and outcomes for HFimpEF tend to be better as compared with persistent HFrEF. Patients with HFimpEF have significantly lower rates of mortality, cardiac hospitalizations, all-cause hospitalizations, and composite events.^4–9^ Moreover, HFimpEF patients have lower rates of cardiac transplantation, LV assist device implantations, and a significant enhancement in health-related quality of life.^10^ However, the underlying mechanisms and factors leading to HFimpEF are not yet fully understood. The improvement in EF seen in HFimpEF can be because of the use of evidence-based medical therapy, device therapies, spontaneous improvement, or a combination of them.^1,11,12^ Nonetheless, specific patient clinical characteristics or treatments that contribute to HFimpEF are not always clear. Factors such as the etiology of the initial injury, female gender, and non-ischemic co-morbidities have been related to EF improvement and subsequent clinical outcomes.^7^ Echocardiographic data on HFimpEF patients have highlighted an initial better LV dimensions, and diastolic function compared with patients with persistent HFrEF.^8,9^ However, the absence of a widespread consensus on the clinical, laboratory, echocardiographic, and therapy factors associated with EF improvement remains a challenge. A better understanding of those factors could help clinicians predict which patients are at risk of persistent HFrEF or HFimpEF. This can inform and design management strategies for this complex population. Therefore, the objective of this study is to evaluate clinical and longitudinal factors associated with subsequent HFimpEF development.

## Methods

This was a single-center retrospective cohort study conducted at the University of California, Davis Medical Center. The study was approved by the university’s Institutional Review Board, and all data used were de-identified before analysis.

Data was collected through the institution’s electronic health record data warehouse between January 2014 and December 2022. The study population consisted of adult patients (≥18 years) diagnosed with HFrEF. The HFrEF cohort inclusion criteria included International Classification of Diseases (ICD) HF codes (ICD-Ninth Revision or ICD-Tenth Revision), LVEF ≤40%, and brain natriuretic peptide (BNP) ≥100 pg/ml. Diagnostic accuracy was evaluated on a random sub-sample (n = 200) of the cohort and compared with physician chart review. Excluded were patients who had ICD codes for cardiac transplant or LV assist device implantation, those who had only one LVEF value in the electronic medical record, and those with less than a 3-month interval between their first and second evaluation of LVEF.

The demographic information extracted consisted of self-reported gender, race, and ethnicity. Baseline laboratory, echocardiogram, and electrocardiogram characteristics were defined as the first value within 90 days after the first LVEF ≤40% or the first available. Co-morbidities were defined as diagnoses before the first LVEF ≤40% date and validated co-morbidity identification methodologies were used.^13,14^ For this study, we defined guideline-directed medical therapy utilization as the prescription of medications within 6 months before and within 3 months after the first LVEF ≤40% date. Data on medication of reconciliation was used to represent the most complete possible information. The medications included renin-angiotensin-system inhibitor (RASi), *β* blockers, mineralocorticoid receptor antagonists, and sodium-glucose co-transporter 2 inhibitors. The RASi category comprised angiotensin-converting enzyme inhibitor, angiotensin receptor blocker, and angiotensin receptor neprilysin inhibitor. Patients were placed into 1 of 3 dosing categories based on their recorded prescription: either none, <50%, or ≥50% of guideline-directed therapy target dosing.^15^

Longitudinal laboratory, electrocardiogram, and echocardiogram variables were extracted, starting from the first LVEF ≤40% until the last available data point on follow-up. Data on all-cause mortality was obtained from the clinical data warehouse, with the latest data available within the study timeline being extracted.

The cohort was stratified into 2 groups: one group consisting of persons with HF who had an improvement in EF (HFimpEF) and a second group consisting of persons who did not experience an improvement but maintained persistently reduced EF (persistent HFrEF). We applied the following criterion to define HFimpEF: an initial LVEF of ≤40%, followed by a subsequent LVEF measurement of >40% at least 3 months later, with a minimum absolute improvement of 10%.^1–3^ Those who did not meet these criteria were categorized as patients with persistent HFrEF.

End points were analyzed in a time-to-event fashion. The primary end point was the development of HFimpEF, and all-cause mortality was the secondary end point. For patients who did not experience HFimpEF, the last LVEF captured was considered the last follow-up (so censoring). For the mortality outcome analysis, the last LVEF was considered the last follow-up.

Descriptive statistics were used to summarize baseline characteristics. Between-group comparisons were performed using Student *t* test or Wilcoxon signed-rank test for continuous variables, and chi-square or Fisher’s exact test for categorical variables as appropriate. In a complete-case analysis fashion, we first conducted univariate Cox regression analyses for each variable for the HFimpEF primary end point. Variables with p <0.20 in univariate analyses were included in the multivariate Cox regression model. The final multivariate model was selected using backward-elimination with a retention threshold of p <0.05. For the multivariate model, collinearity between variables was tested using correlation analyses, with a correlation coefficient threshold of *r* = ±0.50. The robustness of the results and missing data were evaluated by performing a sensitivity analysis with multiple imputation (*m* = 50), followed by pooling the derived parameter estimates and associated standard errors. Kaplan–Meier survival curves and the log-rank test were used to compare overall survival (secondary end point) between the HFimpEF and persistent HFrEF groups. The trajectories of echocardiogram and laboratory parameters over time were plotted using penalized B-spline curves. Linear mixed models for longitudinal data were performed to assess changes between groups over time. Analyses were performed using SAS software version 9.4 (SAS institute, Cary, North Carolina).

## Results

**Figure 1.**
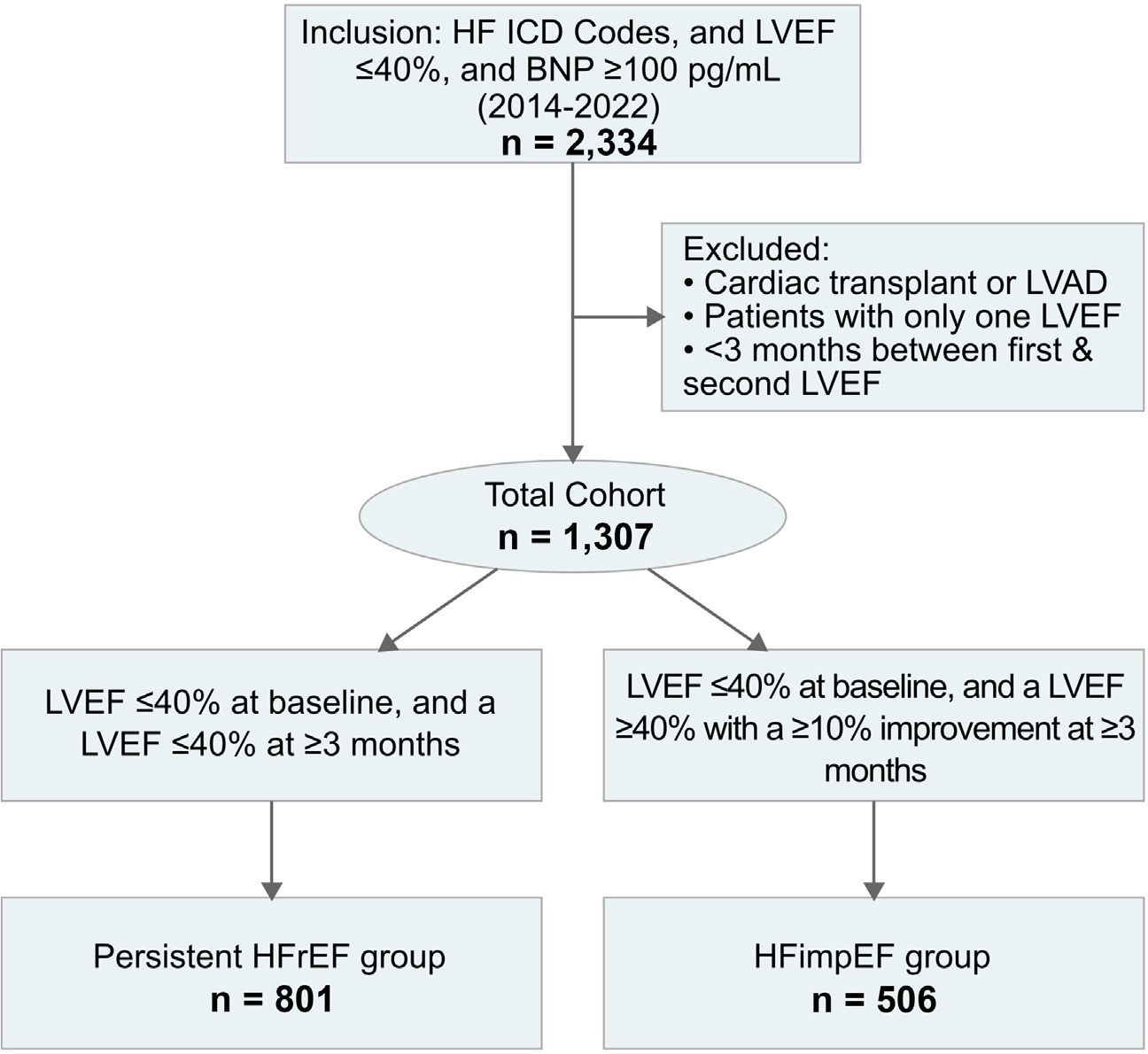
Cohort identification workflow. LVAD = left ventricular assist device.

The cohort identification criteria had a specificity of 0.96 and a sensitivity of 0.60 as compared with physician chart review (Supplementary Table 1). Patients were followed for a median of 16.3 months (interquartile range 8.0 to 30.6). Over the course of follow-up, a total of n = 506 (38.7%) patients developed HFimpEF (50% within the first year), whereas n = 801 (61.3%) had persistent HFrEF (Figure 1, Supplementary Figure 1).

Between-group comparisons of patient baseline characteristics are listed in Table 1. The HFimpEF group consisted of older patients with a higher percentage of females as compared with the persistent HFrEF group. Additionally, the HFimpEF group exhibited a higher heart rate, body mass index, higher prevalence of hypertension and atrial fibrillation, whereas displaying a lower prevalence of ischemic heart disease. The HFimpEF group also had lower BNP and LV internal dimension values, along with higher LVEF, posterior wall, and interventricular septum thickness. The guideline-directed therapy utilization of RASi and *β* blockers at ≥50% target doses was more frequent in the HFimpEF group. However, the persistent HFrEF group displayed an overall higher frequency in the use of RASi and *β* blockers.

**Figure 2.**
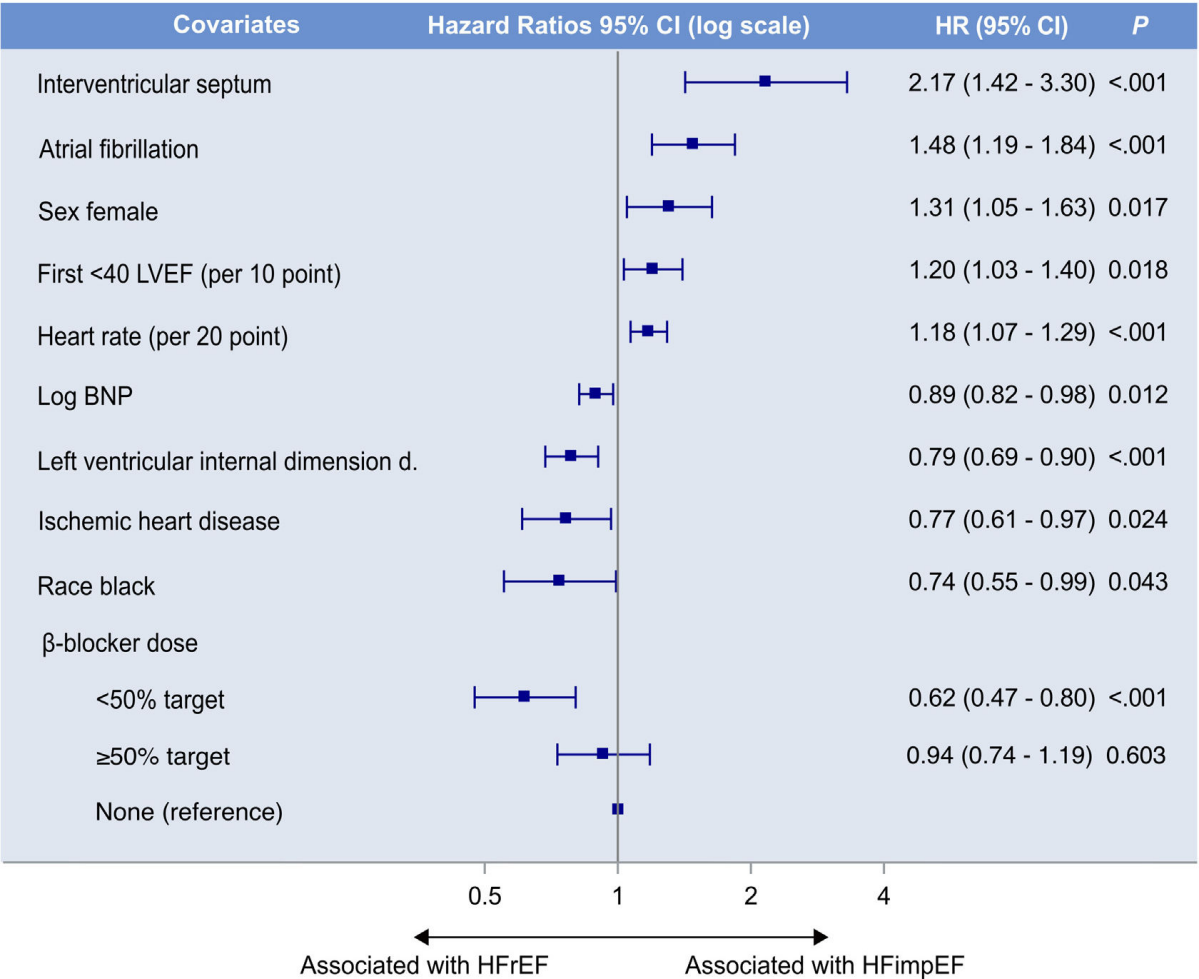
Summary of the multivariate Cox regression model for the HFimpEF primary outcome. A backward-elimination algorithm was utilized to select the final multivariable model, with a retention threshold of p ≤0.05. See Table 2 for details. CI = confidence interval.

Results derived from the univariate analyses and subsequent multivariate Cox regression model for the primary HFimpEF end point are listed in Table 2. In the multivariate model, significant (p <0.05) baseline characteristics associated with HFimpEF included female gender, atrial fibrillation, elevated heart rate, higher first LVEF, and increased thickness of the interventricular septum thickness at end-diastole (IVSd). Covariates associated with persistent HFrEF included Black race, ischemic heart disease, higher levels of BNP, increased LV internal dimension (LVID) at end-diastole and using *β* blockers at <50% of the target dose (results summarized in Figure 2). The HFimpEF multivariate model had an acceptable predicting accuracy with a C-statistic of 0.68. The sensitivity analyses produced similar results (i.e., all predictors retained statistical significance). However, Black race did not reach significance levels (p >0.05), suggesting a weak association with the outcome (Supplementary Table 2)

**Figure 3.**
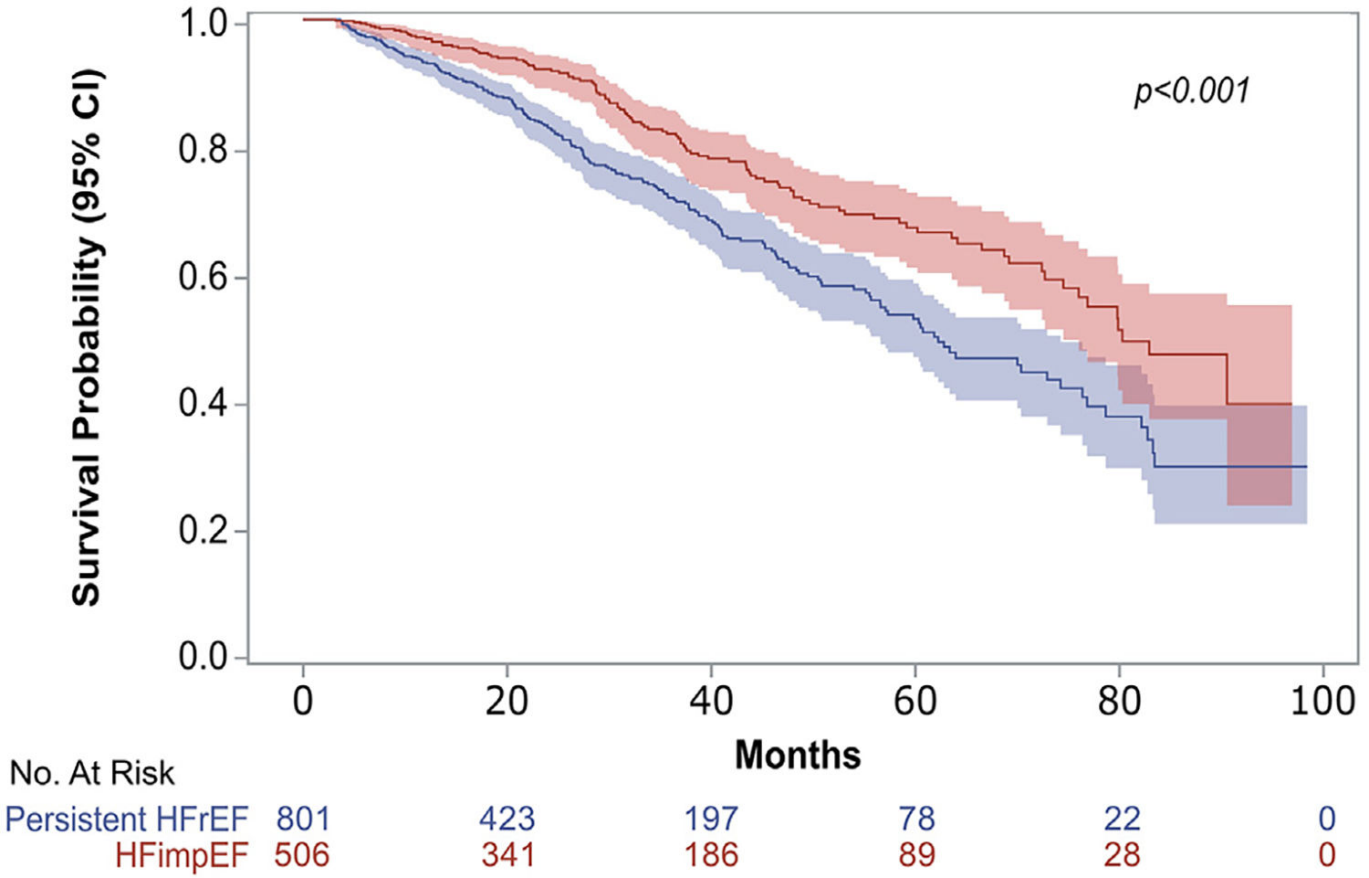
Kaplan-Meier survival curve for all-cause mortality (secondary outcome) by persistent HFrEF vs. HFimpEF group. CI = confidence interval.

For the secondary end point of all-cause mortality, the HFimpEF group had a median survival of 80 months (95% confidence interval: 74.4 to 93.7), whereas the persistent HFrEF group had a median survival of 62 months (95% confidence interval: 55.6 to 74.2). Figure 3 depicts the Kaplan-Meier survival curves, indicating a significantly higher survival in the HFimpEF group (log-rank p <0.001).

**Figure 4.**
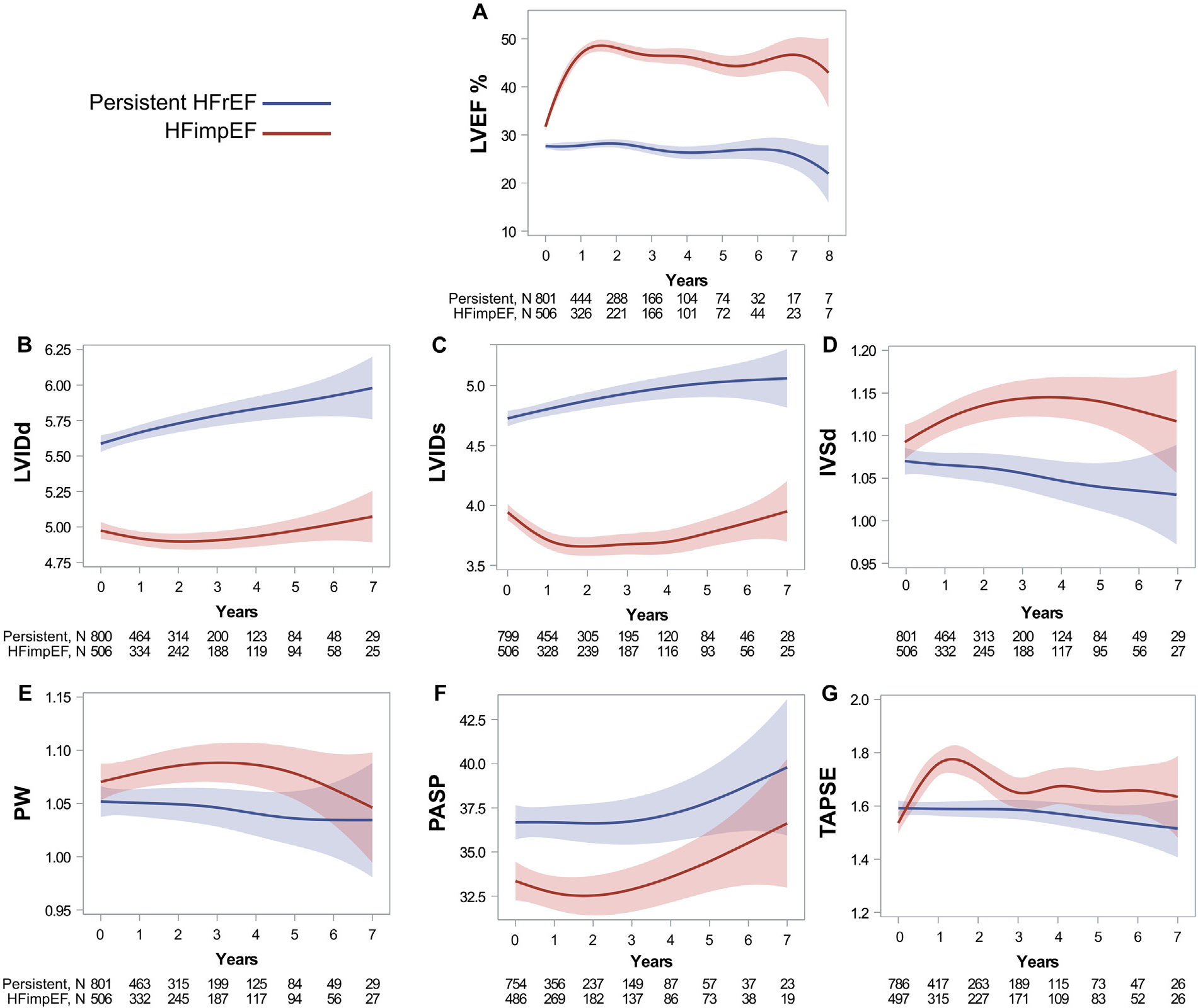
Longitudinal trend of echocardiogram parameters. Data presented as mean (CI) and time as years using penalized B-spline curves. In linear mixed models, LVEF, IVSd, LVIDd, LVIDs, and TAPSE parameters exhibited significant changes over time (p <0.001). Only PW and PASP exhibited slight changes, with the same initial trend over time (p = 0.397 and p = 0.067 respectively).

Echocardiogram trajectories are depicted in Figure 4. For the HFimpEF group, there was a marked LVEF improvement within the first year, with a trend to decline over time. Conversely, the persistent HFrEF group showed a continuous trend in LVEF decline. Overall, the time-dependent echocardiogram parameters demonstrated that the HFimpEF group had an increasing trend in LVEF, IVSd, posterior wall, and tricuspid annular plane systolic excursion (TAPSE). Meanwhile, a decreasing trend was observed in LVID at end-diastole, LVID at end-systole, and pulmonary artery systolic pressure (PASP). Linear mixed models showed statistically significant (p <0.001) changes between groups over time for LVEF, IVSd, LVID at end-diastole, LVID at end-systole, and TAPSE. However, posterior wall and PASP showed minimal longitudinal changes, following the same initial trend (p = 0.397 and p = 0.067, respectively).

**Figure 5.**
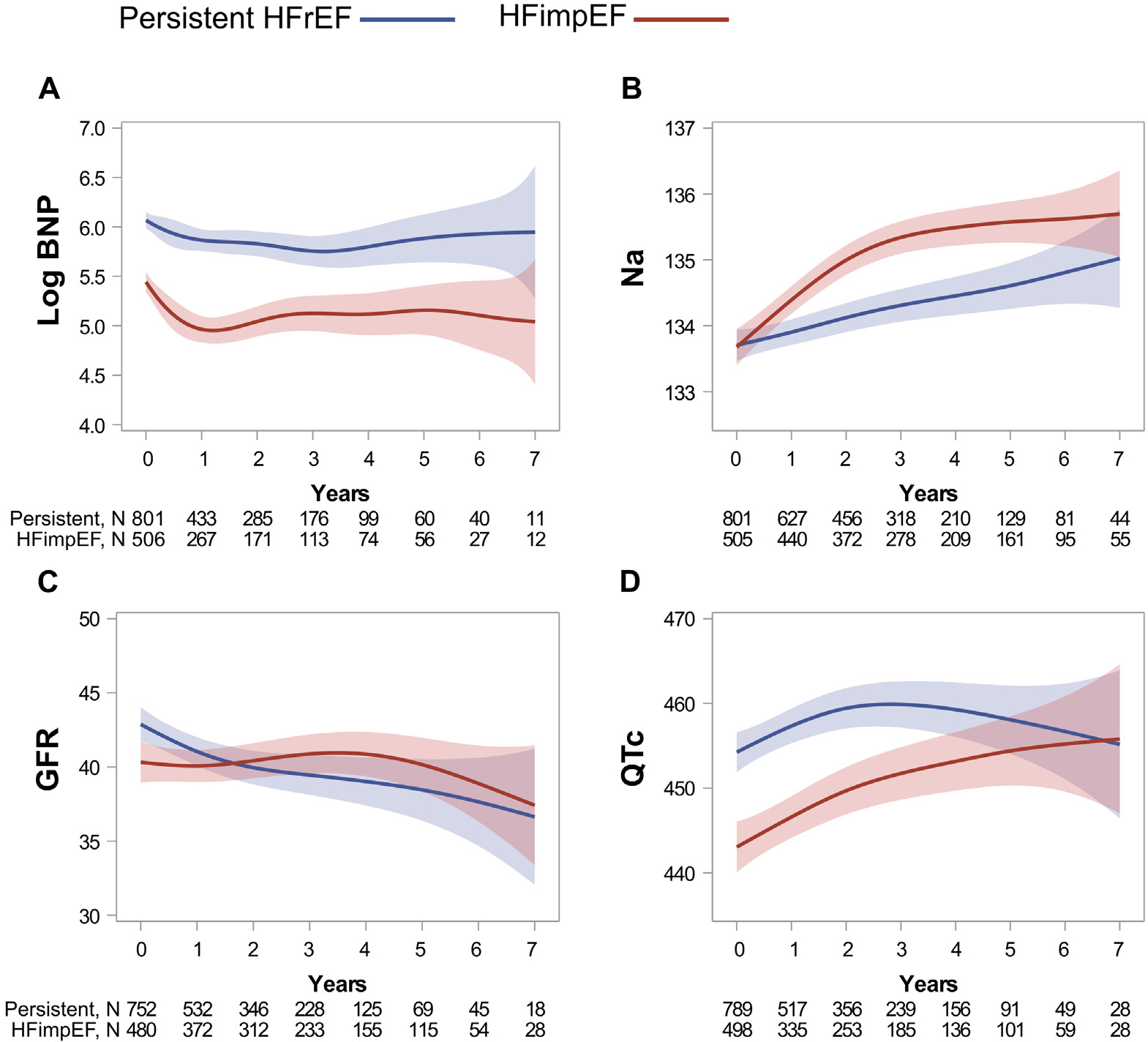
Longitudinal trend of labs and QTc parameters. Data presented as mean (CI) and time as years using penalized B-spline curves. In linear mixed models, eGFR and sodium showed significant changes over time (p <0.001). Contrarily, BNP, and QTC presented the same longitudinal trend without significant changes over time (p = 0.083 and p = 0.129 respectively). eGFR = estimated glomerular filtration rate; Na = sodium; QTc = corrected QT interval for heart rate.

The biomarkers and corrected QT trajectories are shown in Figure 5. The HFimpEF group demonstrated significantly increased levels of sodium and estimated glomerular filtration rate (p <0.001) over time. However, after year 4, estimated glomerular filtration rate values seemed to demonstrate a decline. At the same time, BNP and corrected QT values showed slight variations over time, following the same initial trend (p = 0.083 and p = 0.129, respectively).

## Discussion

This retrospective HFrEF cohort was conducted to evaluate clinical and longitudinal characteristics associated with HFimpEF. In our HFrEF cohort, it was found that 38.7% of patients transitioned to HFimpEF and 50% of these patients made this transition within their first follow-up year. The HFimpEF Cox regression model identified baseline covariates of persistent or improved HFrEF, which included gender, race, co-morbidities, echocardiographic parameters, and BNP. Thus, opening venues for HFrEF improvement risk stratification. Moreover, the HFimpEF group demonstrated distinct echocardiographic and laboratory trajectories and improved survival rates as compared with patients in the persistent HFrEF group.

The baseline characteristics of our HFimpEF cohort consisted of patients who were more likely to be older, female, and with increased prevalence of co-morbidities such as hypertension and atrial fibrillation. This suggests a non-ischemic profile, which may increase the likelihood of LVEF improvement. In contrast, the persistent HFrEF group demonstrated a higher prevalence of ischemic heart disease, higher BNP levels, and less favorable LV geometry and function (LV internal dimensions, posterior wall, IVSd, and LVEF). These characteristics are consistent with previous studies.^4–9^ Lastly, the persistent group exhibited worse echocardiogram and BNP profiles, and they were prescribed RASi and *β* blockers more often. This suggests that these treatments were more frequently prescribed to patients with more severe HF.

In the multivariate Cox regression analysis, multiple characteristics were associated with HFimpEF (Table 2 and summarized in Figure 2). Of note, in contrast with previous research,^7,8^ associations with HFimpEF were investigated in a time-to-event fashion. These findings could aid in the stratification of patients at risk for developing persistent HFrEF.

Previous research has highlighted that female biologic gender is associated with improvement in LVEF.^7,8^ This association was also observed in our multivariate model. Additionally, Black race was related with persistent HFrEF. Previous studies have highlighted that Black patients have an increased susceptibility to structural cardiac remodeling that is more associated with worse systolic function.^16–18^ Although, in our sensitivity analysis, Black race resulted in a weak association, these findings support the need for further exploration of the racial disparities underpinning HF in Black patients. Consistent with other HFimpEF studies,^7,8^ patients with atrial fibrillation had an increased likelihood of improving their EF. It is possible that effective medical or device therapy for controlling atrial fibrillation contributes to this result. In contrast, patients with ischemic heart disease have compromised blood flow to the cardiac muscle with subsequent permanent and irreversible cardiac damage. This type of injury may explain why these patients had a decreased probability of EF improvement, as has been reported in other studies.^7,8^

An unfavorable LV geometry and function (LVID at end-diastole, IVSd, and LVEF) at the time of diagnosis would be less likely to improve from a mechanistic standpoint. Similarly, elevated levels of BNP can also be a surrogate of unfavorable cardiac function. This may explain the predictive value of these echocardiograms and BNP markers found in our HFimpEF model.

As listed in Table 1, *β* blockers at <50% of target doses were more frequently prescribed to patients with more severe HF, the persistent HFrEF group. This explains the apparent inverse relation between *β* blockers at <50% of target dose and the HFimpEF outcome. Of note, the retrospective nature of our study does not establish causality. Instead, our findings should be interpreted as associations rather than direct causes and effects.

The RASi and *β* blockers at ≥50% target doses were more common in the HFimpEF group (Table 1). However, in the multivariate analysis neither were independently associated with HFimpEF (Table 2). These findings are consistent with other studies that have reported a lack of association between guideline-directed therapy and the definition of HFimpEF.^7,8^ One possible explanation for this could be the enhancement in LVEF by guideline-directed therapy in real-world clinical settings is insufficient to meet the established definition criteria for HFimpEF.^3^ Furthermore, guideline-directed therapy is frequently underused and underdosed in health care systems,^19^ as observed in this study.

Lastly, the persistent HFrEF group was also associated with increased utilization of *β* blockers at <50% of target along with lower heart rate and higher prevalence of ischemic heart disease. Taken together, this constellation of features delineates a profile of ischemic injury, frequent use of *β* blockers, and lower heart rate related to persistently reduced EF on follow-up.

In the present study, patients in the HFimpEF group demonstrated longer survival rates than their persistent HFrEF counterparts. These results are supported by previous studies demonstrating improved survival rates in patients with HFimpEF.^4–8^ This finding highlights the importance of identifying patients who are at risk of having persistent HFrEF and the need to design and implement interventions to impact outcomes.

The longitudinal echocardiographic analysis of the HFimpEF group versus the persistent HFrEF group demonstrated contrasting differences in longitudinal trajectories, most notably, marked LVEF improvement within the first year for the HFimpEF group. Moreover, the HFimpEF group also exhibited increasing trends in LVEF, IVSd, posterior wall, and TAPSE and decreasing trends in LVID at end-diastole, LVID at end-systole, and PASP over time. These cardiac geometric and functional changes are indicative of reverse remodeling in the HFimpEF group and remodeling in the HFrEF group.

The values for BNP and corrected QT exhibited small variations over time, following the same initial longitudinal pattern over time between groups. This points toward a persistent electrical remodeling and the persistence of initial BNP levels over time in both groups. Lastly, the HFimpEF group also experienced an increase in sodium and estimated glomerular filtration rate over time, suggesting an improved HF leading to renal function enhancement, a decrease in neurohormonal activation, and improvement in renal retention of sodium and water. However, despite initial improvements, there was a long-term decline in estimated glomerular filtration rate over time (after 4 or more years of follow-up).

The HFimpEF Cox regression model in the present study has the potential to inform stratification of HFrEF patients who are at risk of experiencing persistent HFrEF at baseline. Early identification of patients at risk for persistent HFrEF creates opportunities to design and implement targeted quality-of-care strategies for guideline-directed therapy, device therapy, timely referrals to advanced HF care, and monitoring strategies to impact HF outcomes. However, although the model yielded a promising C-statistic of 0.68 given the complexity of the data at hand, validation is required before the model can be implemented for clinical purposes. Although prospective randomized studies are ideal for such validation, external and internal validation methodologies can determine reproducibility and generalizability. Additionally, machine learning models could potentially offer more accuracy.

The possible underlying mechanisms for the clinical outcome post-insult are likely multifactorial in nature. Cardiomyocyte loss because of apoptosis, necrosis, or pyroptosis is triggered by multiple factors including ischemia, pressure-overload, and mitochondrial dysregulation inducing critical transcriptional factors, inflammatory cytokines, and growth factors such as nuclear factor kappa B, tumor necrosis factor, and transforming growth factor-beta, leading to further cardiac fibrosis and cell death.^20–23^ The underlying co-morbidities, lifestyle modification, and the lack of optimal guideline-directed therapy can significantly alter the underlying mechanisms, leading to adverse electrical and structural remodeling.^24,25^ Furthermore, the intricate balance of intracellular calcium and sodium levels in cardiomyocytes, controlled by sarcoplasmic reticulum calcium ATPase, sodium/calcium exchanger, and sodium and calcium channels, may contribute not only to HF phenotype including cardiac arrhythmias but also mechanistically through transcriptional activation and posttranslational modification.^26–28^ To further decipher the myriads of mechanistic underpinnings contributing to HF progression, multiple animal HF models can be utilized with the ultimate goal of developing novel therapeutic targets.^29^ Nevertheless, a comprehensive exploration into these mechanisms remains an area for future research.

This study has limitations that should be considered when interpreting the results. First, the cohort was derived from a single center, which may limit the generalizability of our findings to other populations and healthcare settings. Second, the observational and retrospective nature of our study precludes the identification of clear causal etiologies of HF, and we cannot establish causal inferences between the identified predictors and the development of HF improvement. Our findings should be interpreted as associations rather than direct causes and effects. Prospective studies would be needed to delve deeper into etiologies and causal inferences. Additionally, despite adjusting for multiple variables in the multivariate Cox regression analysis, residual confounding because of unmeasured factors cannot be ruled out. Finally, cause-specific mortality analysis is lacking as our institution does not routinely gather cause-specific mortality information.

In conclusion, the present study demonstrates that 38.7% of patients transitioned to HFimpEF in our HFrEF cohort. Key baseline characteristic predictors in an HFimpEF Cox regression model included gender, co-morbidities, echocardiographic measurements, and BNP. If further validated to determine reproducibility, this model could potentially contribute to risk stratification assessment for persistent or improved HFrEF. Early risk stratification enables design and implementation of targeted interventions for quality-of-care improvement impacting HF outcomes. Moreover, the HFimpEF group exhibited better survival rates, and distinct longitudinal echocardiogram and laboratory trajectories when compared with persistent HFrEF.

## Supplementary Material

1

## Figures and Tables

**Table 1:** Patient baseline characteristics at the time of first LVEF ≤40%

	Median (IQR) or No. (%)				
Characteristics	All (N=1307)	Persistent HFrEF (N=801)	HFimpEF (N=506)	*P*-Value*	*n*

Age, years	65(55–75)	63 (54–74)	66 (58–76)	**<.001**	1307
**Sex**				**<.001**	1307
Male	887 (67.9)	582 (72.7)	305 (60.3)		
**Race**				0.157	1307
White	742 (56.8)	439 (54.8)	303 (59.9)		
Black	229 (17.5)	155 (19.4)	74 (14.6)		
Asian	86 (6.6)	49 (6.1)	37 (7.3)		
Native American/Hawaiian	41 (3.1)	25 (3.1)	16 (3.2)		
Other	200 (15.3)	129 (16.1)	71 (14.0)		
Unavailable	9 (0.7)	4 (0.5)	5 (1.0)		
**Ethnicity**				0.910	1307
Hispanic	173 (13.2)	108 (13.5)	65 (12.9)		
Non-Hispanic	1128 (86.3)	689 (86.0)	439 (86.8)		
Unavailable	6 (0.5)	4 (0.5)	2 (0.4)		
Heart rate, b.p.m.	88(75–102)	86 (73–100)	90 (77–105)	**<.001**	1306
**Blood pressure, mm Hg**					
Systolic	127(112–143)	127 (112–142)	127 (112–145)	0.885	1307
Diastolic	77 (67–90)	76 (67–90)	78 (67–90)	0.345	1307
MAP	94.3 (82.7–106.7)	93.7 (82.7–106)	95.3 (83.3–107)	0.395	1307
Weight, Kg	83.5 (71.2–101.1)	83.1 (70.6–98.7)	84.1 (72.4–103.5)	0.104	1306
Body mass index	28.2 (24.5–32.9)	27.9 (24.2–32.2)	28.7 (24.9–33.7)	**0.024**	1172
**Medical History**					1295
Hypertension	967 (74.7)	575 (72.4)	392 (78.2)	**0.019**	
Diabetes	568 (43.9)	347 (43.7)	221 (44.1)	0.885	
Hyperlipidemia	574 (44.3)	330 (41.6)	244 (48.7)	**0.012**	
Ischemic heart disease	478 (36.9)	318 (40.1)	160(31.9)	**0.003**	
Atrial fibrillation	436 (33.7)	231 (29.1)	205 (40.9)	**<.001**	
Chronic kidney disease	340 (26.3)	210 (26.5)	130 (25.9)	0.842	
**Laboratory**					
BNP, pg/mL	623 (265–1330)	750 (323–1521)	451.5 (209–926)	**<.001**	1307
NT-proBNP, pg/mL	1110(326–2914)	1242(431–3339)	874 (218–2852)	0.214	148
Sodium, mEq/L	138 (135–139)	138 (135–139)	138 (136–140)	0.067	1306
Potassium, mEq/L	4 (3.7–4.4)	4 (3.7–4.4)	4 (3.7–4.4)	0.813	1306
Creatinine, mg/dL	1.2 (0.9–1.5)	1.2 (0.9–1.5)	1.2 (0.9–1.5)	0.952	1306
eGFR, mL/min/1.73 m2	56 (46–60)	57 (47–61)	55 (45–60)	**0.048**	1232
**Echocardiogram**					
LVEF %	30 (25–40)	30 (20–35)	35 (30–40)	**<.001**	1307
IVSd	1.2(1–1.3)	1.2 (1–1.3)	1.2 (1.1–1.4)	**<.001**	1307
LVIDd	5.6 (5.1–6.2)	5.8 (5.3–6.4)	5.4 (4.9–5.8)	**<.001**	1306
LVIDs	4.7 (4.1–5.4)	4.9 (4.3–5.6)	4.4 (3.8–4.9)	**<.001**	1305
PASP	41 (30.5–50)	41.5 (31.2–50.4)	40 (29.7–49.7)	0.293	1240
PW	1.2(1–1.3)	1.1 (1–1.3)	1.2 (1–1.3)	**<.001**	1307
TAPSE	1.8 (1.4–2.1)	1.8 (1.4–2.1)	1.8 (1.4–2.1)	0.818	1283
**Electrocardiogram**					
QTc, ms	498 (472–528)	499 (474–528)	495.5 (470–527)	0.161	1287
**GDMT & target dose^†^**					1059
RASi^c^				**0.004**	
none	188(17.8)	109 (16.9)	79 (19.1)		
<50% target	435 (41.1)	291 (45.1)	144 (34.9)		
≥50% target	436(41.2)	246(38.1)	190 (46)		
*β*-Blocker				**<.001**	
none	406 (38.3)	214 (33.1)	192 (46.5)		
<50% target	349 (33)	255 (39.5)	94 (22.8)		
≥50% target	304 (28.7)	177 (27.4)	127 (30.8)		
MRA				0.654	
none	694 (65.5)	430 (66.6)	264 (63.9)		
<50% target	2 (0.2)	1 (0.2)	1 (0.2)		
≥50% target	363 (34.3)	215 (33.3)	148 (35.8)		
SGLT2i	70 (6.6)	48 (7.4)	22 (5.3)	0.179	
GDMT Triple therapy	178(16.8)	120(18.6)	58 (14)	0.054	
GDMT Triple, ≥50% target	60 (5.7)	34 (5.3)	26 (6.3)	0.479	

* P-Values comparing HFimpEF vs. persistent HFrEF.

† Target doses of guideline directed medical therapy (GDMT) as recommended in treatment guidelines.

§ RASi consisted of angiotensin-converting enzyme inhibitor, angiotensin receptor blocker, angiotensin receptor neprilysin inhibitor.

BNP = B-type natriuretic peptide; eGFR = estimated glomerular filtration rate; GDMT = guideline-directed medical therapy; IVSd= Interventricular septum thickness at end-diastole; LVEDd = left ventricle end diastolic diameter; LVEF = left ventricular ejection fraction; LVIDd = left ventricular internal dimension at end-diastole; LVIDs = left ventricular internal dimension at end-systole; MAP = mean arterial pressure; MRA = mineralocorticoid receptor antagonist; NT-proBNP = N-terminal (NT)-pro hormone BNP; PASP = pulmonary artery systolic pressure; PW = left ventricular posterior wall; QTc = QT corrected for heart rate; RASi = renin-angiotensin-system inhibitor; SGLT2i = Sodium glucose co-transporter 2 inhibitors; TAPSE = tricuspid annular plane systolic excursion.

**Table 2:** Cox regression analyses for the of HFimpEF, primary endpoint

Variable	Univariate models		Multivariate model*	
	HR (95% CI)	*P* value	HR (95% CI)	*P* value

Age at start, years	1.01 (1.01–1.02)	<.001	.	.
Female	1.54(1.29–1.84)	<.001	1.31 (1.05–1.63)	**0.017**
Race	.	.	.	.
Asian	1.12(0.80–1.58)	0.504	.	.
Black^†^	0.76 (0.59–0.98)	0.032	0.74 (0.55–0.99)	**0.043**
Native American/Hawaiian	1.18(0.71–1.94)	0.531	.	.
Other	0.85 (0.66–1.10)	0.227	.	.
Unavailable	1.16(0.48–2.80)	0.746	.	.
White	Reference	.	.	.
Atrial fibrillation	1.54(1.29–1.84)	<.001	1.48(1.19–1.84)	**<.001**
Hypertension	1.28(1.03–1.58)	0.024	.	.
Ischemic heart disease	0.81 (0.67–0.98)	0.028	0.77 (0.61–0.97)	**0.024**
Hyperlipidemia^‡^	1.24(1.04–1.48)	0.016	.	.
Heart rate (per 20-point increase)	1.15 (1.06–1.25)	<.001	1.18(1.07–1.29)	**<.001**
*β*-blocker	.	.	.	.
≥50%	0.82 (0.66–1.02)	0.075	0.94 (0.74–1.19)	0.603
<50%	0.49 (0.38–0.63)	<.001	0.62 (0.47–0.80)	**<.001**
None	Reference	.	.	.
Log BNP^§^	0.87 (0.81–0.93)	<.001	0.89 (0.82–0.98)	**0.012**
GFR	0.99 (0.98–1.00)	0.005	.	.
First LVEF ≤40 (per 10-point increase)	1.46(1.30–1.64)	<.001	1.20(1.03–1.40)	**0.018**
LVIDd^‡^	0.65 (0.58–0.72)	<.001	0.79 (0.69–0.90)	**<.001**
IVSd^‡^	2.12(1.52–2.94)	<.001	2.17(1.42–3.30)	**<.001**

* Predictors from the univariate analysis that showed a p-value of ≤20 were subsequently incorporated into a multivariate analysis. A backward-elimination algorithm was utilized to select the final multivariable model, with a retention threshold ofp≤05. The multivariate model had a C-statistic of 0.68.

† In the multivariate analysis, race was categorized as black or other.

‡ Hyperlipidemia was omitted in the multivariate model due to possible overlapping effect with ischemic heart disease. PW and LVIDs were excluded due to the correlation coefficient >0.5 with IVSd and LVIDd respectively.

§ The variable has been log-transformed.

BNP = B-type natriuretic peptide; eGFR = estimated glomerular filtration rate; GDMT = guideline-directed medical therapy; IVSd = Interventricular septum thickness at end-diastole; LVEDd = left ventricle end diastolic diameter; LVEF = left ventricular ejection fraction; LVIDd = left ventricular internal dimension at end-diastole; LVIDs = left ventricular internal dimension at end-systole; MAP = mean arterial pressure; MRA = mineralocorticoid receptor antagonist; NT-proBNP = N-terminal (NT)-pro hormone BNP; PASP = pulmonary artery systolic pressure; PW= left ventricular posterior wall; QTc = QT corrected for heart rate; RASi = renin-angiotensin-system inhibitor; TAPSE = tricuspid annular plane systolic excursion.
