# Supplementary material for: Clinical, Echocardiographic, and Longitudinal Characteristics Associated With Heart Failure With Improved Ejection Fraction
Source: Am J Cardiol. Author manuscript; Available in PMC 2024 Feb 15. (PMC10869234; doi:10.1016/j.amjcard.2023.10.086)
Supplement: 1 [file NIHMS1960304-supplement-1.docx]

**SUPPLEMENTARY MATERIAL**


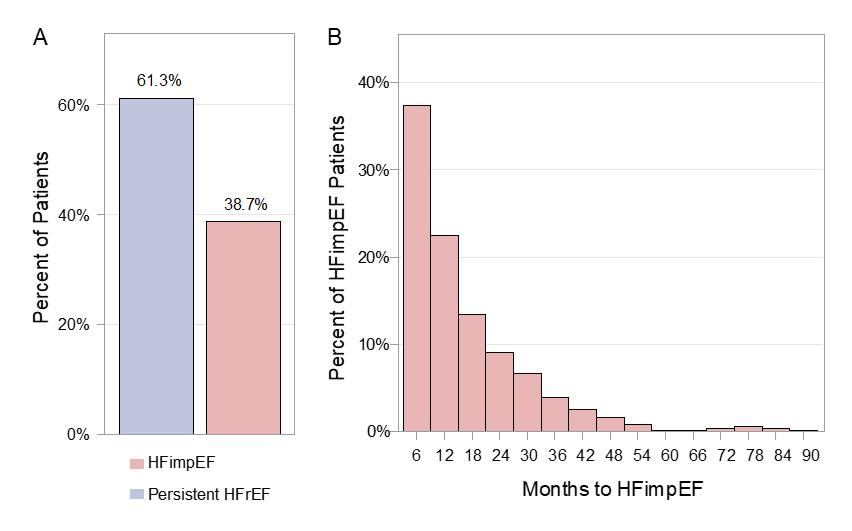


**Figure S1**. (**A**) Proportion of patients from the HFrEF cohort (n=1307) who either transitioned to HFimpEF (n=506, 38.7%) or persistent HFrEF (n=801, 61.3%). (**B**) Among the HFimpEF group (n=506), 50% reached the improvement definition within the first 12 months. Histogram representing in the Y-axis the proportion of patients and X-axis representing time to reach the HFimpEF definition.

| **Table S1.** Cohort diagnostic performance vs. chart review. | | | | |
| --- | --- | --- | --- | --- |
| **Algorithm: ICD codes + LVEF ≤40 + BNP ≥100** | | | | |
| AUC | Sensitivity (CI) | Specificity (CI) | PPV (CI) | NPV (CI) |
| 0.92 | 0.60 (0.48-0.72) | 0.96 (0.91-0.99) | 0.90 (0.80-0.99) | 0.79 (0.72- 0.87) |
| Abbreviations: AUC, area under the curve; PPV, positive predictive value; NPV, negative predictive value; CI, confidence interval; ROC, Receiver Operator Characteristic | | | | |

| **Table S2.** Cox regression analyses with Multiple imputation (*m*=50) for the of HFimpEF primary endpoint | | | | | | | |
| --- | --- | --- | --- | --- | --- | --- | --- |
|  | **Multivariate model (no MI)** | | |  | | **Multivariate model with MI^a^** | |
| **Variable** | **HR (95% CI)** | | ***P* value** |  | **HR (95% CI)** | | ***P* value** |
| Female | | 1.31 (1.05-1.63) | **0.017** |  | 1.35 (1.11-1.64) | | **0.002** |
| Race | | . | . |  | . | | . |
| Black^b^ | | 0.74 (0.55-0.99) | **0.043** |  | 0.80 (0.62-1.04) | | 0.096 |
| Other | | Reference | . |  | . | | . |
| Atrial fibrillation | | 1.48 (1.19-1.84) | **<.001** |  | 1.51 (1.25-1.82) | | **<.001** |
| Ischemic heart disease | | 0.77 (0.61-0.97) | **0.024** |  | 0.77 (0.63-0.94) | | **0.01** |
| Heart rate (per 20-point increase) | | 1.18 (1.07-1.29) | **<.001** |  | 1.16 (1.07-1.26) | | **<.001** |
| β-blocker | | . | . |  | . | | . |
| ≥50% | | 0.94 (0.74-1.19) | 0.603 |  | 0.97 (0.76-1.23) | | 0.786 |
| <50% | | 0.62 (0.47-0.80) | **<.001** |  | 0.70 (0.54-0.89) | | **0.004** |
| None | | Reference | . |  | . | | . |
| Log BNP^d^ | | 0.89 (0.82-0.98) | **0.012** |  | 0.90 (0.83-0.97) | | **0.006** |
| First LVEF ≤40 (per 10-point increase) | | 1.20 (1.03-1.40) | **0.018** |  | 1.21 (1.05-1.38) | | **0.006** |
| LVIDd^c^ | | 0.79 (0.69-0.90) | **<.001** |  | 0.79 (0.70-0.89) | | **<.001** |
| IVSd^c^ | | 2.17 (1.42-3.30) | **<.001** |  | 2.08 (1.45-2.98) | | **<.001** |
| ^a^Predictors from the main multivariate analysis that showed a *p* value of ≤.05 were subsequently incorporated into a multivariate analysis with multiple imputation (*m*=50), followed by pooling the derived parameter estimates and associated standard errors.  ^b^In the multivariate analysis, race was categorized as black or other.  ^c^Hyperlipidemia was omitted in the multivariate model due to possible overlapping effect with ischemic heart disease. PW and LVIDs were excluded due to the correlation coefficient >0.5 with IVSd and LVIDd respectively.  ^d^The variable has been log-transformed.  **Abbreviations:** BNP= B-type natriuretic peptide; eGFR= estimated glomerular filtration rate; GDMT= guideline-directed medical therapy; IVSd= Interventricular septum thickness at end-diastole; LVEDd= left ventricle end diastolic diameter; LVEF= left ventricular ejection fraction; LVIDd= left ventricular internal dimension at end -diastole; LVIDs= left ventricular internal dimension at end -systole; MAP= mean arterial pressure; MRA= mineralocorticoid receptor antagonist: MI= multiple imputation; NT-proBNP, N-terminal (NT)-pro hormone BNP; PASP= pulmonary artery systolic pressure; PW= left ventricular posterior wall; QTc= QT corrected for heart rate; TAPSE= tricuspid Annular Plane Systolic Excursion. | | | | | | | |
